# From byte to bench to bedside: molecular dynamics simulations and drug discovery

**DOI:** 10.1186/s12915-023-01791-z

**Published:** 2023-12-29

**Authors:** Mayar Ahmed, Alex M. Maldonado, Jacob D. Durrant

**Affiliations:** Department of Biological Sciences, University of Pittsburgh, Pittsburgh, PA USA

## Abstract

**Molecular dynamics (MD) simulations and computer-aided drug design (CADD) have advanced substantially over the past two decades, thanks to continuous computer hardware and software improvements. Given these advancements, MD simulations are poised to become even more powerful tools for investigating the dynamic interactions between potential small-molecule drugs and their target proteins, with significant implications for pharmacological research.**

## From trial and error to rational drug design

Throughout most of human history, drug discovery relied on trial and error. Modern structural biology has revolutionized the field by enabling rational drug design. This approach uses the molecular structures of disease-implicated targets (typically proteins) to guide the identification and optimization of small-molecule ligands—initial hits that can be further developed into drugs. Structure-based computeraided drug design (CADD) further augments rational design by using computational methods to drastically reduce the physical experiments required for hit identification, making early-stage drug discovery more cost-effective and efficient.

Traditional CADD techniques focus on static protein structures. But proteins are highly dynamic in solution, and ligand-binding pockets often sample many pharmacologically relevant shapes (i.e., conformations). A given small-molecule ligand may bind to and stabilize only a subset of conformations that complement its shape and specific arrangement of interacting functional groups. Moreover, different ligands often stabilize distinct yet equally valid pocket conformations. CADD methods that exclusively consider a single pocket conformation thus run the risk of overlooking potential ligands that may bind to alternative conformations.

Molecular dynamics (MD) simulations have emerged as valuable tools for investigating the conformational diversity of ligand binding pockets. These simulations approximate the complex quantum–mechanical forces that govern atomic motions by representing atoms and bonds as simple spheres connected by virtual springs [1]. CADD researchers routinely use MD simulations to unveil pharmacologically relevant conformational changes, allosteric mechanisms, and binding-pocket dynamics. In this comment, we provide a concise overview of the intersection between MD simulations and CADD over the past two decades, emphasizing the advancements that have enhanced our understanding of protein flexibility and its profound impact on drug discovery.

## Generating conformational ensembles

To identify structurally diverse small-molecule ligands that bind to a dynamic binding pocket, CADD must account for multiple physiologically relevant pocket conformations. MD simulations are valuable tools for capturing these continuous conformational changes, including the opening and closing of transient druggable subpockets that are challenging to detect experimentally. By clustering the many conformations sampled during an MD simulation, one can generate a condensed yet diverse set of representative pocket conformations, known as a conformational ensemble, for use in subsequent CADD analyses.

### Enriching conformational ensembles by capturing long-timescale dynamics

Longer simulations often reveal more comprehensive conformational ensembles. Short simulations primarily capture rapid molecular events such as local fluctuations, surface sidechain rotations, and fast loop reorientations. In contrast, longer simulations show how slow loop reorientations, buried sidechain rotations, and some allosteric transitions impact binding-pocket geometries, revealing druggable conformations that shorter simulations rarely sample.

Advancements in computer hardware over the past two decades have enabled much longer simulations. Notably, the adoption of graphics processing units (GPUs) has revolutionized the field by dramatically accelerating calculations. Designed initially as highly parallel processors to enhance video game performance, GPUs have been repurposed to accelerate scientific calculations, including those required for faster and more efficient MD simulations. Ever-increasing supercomputer resources have also enabled longer simulations. Supercomputing power is commonly measured in floating-point operations per second (FLOPS). The performance of the world’s top supercomputer in 2000 was 2.4 trillion FLOPs roughly the same as an iPhone 14 Pro. By 2010, this performance had increased to 1.8 quadrillion FLOPS and then to 1.2 quintillion FLOPs in 2023.

These hardware advances now allow researchers to better explore the pharmacological implications of longer timescale dynamics. The first MD simulation was performed in 1977 and captured only 8.8 ps of bovine pancreatic trypsin inhibitor dynamics [2]. It took another 21 years to achieve the first microsecond simulation of a protein in explicit solvent a remarkable 10-million-fold increase in simulation length and since 2010, several millisecond-regime simulations have been reported.

Emerging hardware technologies will soon enable even longer simulations. Many computing tasks benefit from application-specific integrated circuits (ASICs) and custom-designed chips tailored for a specific task rather than general-purpose use. As the demand for accelerated MD simulations grows among academic and industry researchers, we expect the proliferation of ASICs with optimized architectures explicitly designed for MD acceleration, such as those used in the Anton series of supercomputers. Future systems incorporating ASICs, related chips called field-programmable gate arrays (FPGAs), or other specialized hardware could enable routine access to longer, biologically relevant timescales.

Aside from hardware advancements, software advancements have also allowed MD simulations to more thoroughly sample physiologically relevant conformations. Several methods aim to algorithmically improve sampling along pre-defined pathways that connect different conformational states (the “progress coordinate”), but others, such as replica exchange, hyperdynamics, and some machine-learning approaches, require no predefined coordinate.

More recently, some have sought to enhance binding-pocket sampling by coupling MD and AlphaFold [3], a recently developed machine-learning approach for protein structure prediction. MD is critical because AlphaFold and related methods often struggle to position side chains with the accuracy required for effective CADD. MD simulations can correct misplaced side chains, substantially improving subsequent ligand-binding predictions [4]. Modified AlphaFold pipelines also overcome the default implementation’s tendency to converge on a single conformation, making it possible to predict entire conformational ensembles [5]. These multiple conformations can then serve as seeds for short simulations, bypassing the need for long-timescale simulations that would otherwise be required to transition between the conformational states.

Software acceleration has also benefited advanced simulations that capture quantum effects. Classical “sphere-and-spring” MD simulations overlook crucial interactions such as electron correlation, nuclear quantum effects, and electron delocalization. Consequently, classical simulations cannot model chemical reactions, nor can they account for some subtle non-covalent effects that may impact ligand binding. Accurately accounting for these factors requires computationally intensive quantum mechanical (QM) methods such as Kohn–Sham density functional theory (DFT). Machine-learning models trained on millions of DFT calculations have the potential to drastically reduce the computing time required for these calculations. These models learn to predict quantum effects without having to perform the corresponding computationally intensive calculations. Although they enable otherwise intractable QM calculations, these models are still much slower than classical methods. We expect ongoing advancements in computer speed and accuracy to broaden their adoption in the future.

### Enriching conformational ensembles via mesoscale simulations

Simulating protein targets embedded in larger macromolecular complexes or realistic subcellular environments can also help identify more complete ensembles of physiologically relevant pocket conformations. These simulations better account for the impact that interactions with macromolecular partners have on binding-pocket geometries. Over the past two decades, researchers have made remarkable progress in simulating increasingly larger systems. The first atomistic MD simulation performed in 1977 had fewer than 1000 atoms [2]; by 2002, this count increased over 100-fold (~ 100,000 atoms), and by 2017, it had increased to ~ 1 million atoms. In recent years, several simulations have surpassed the 100-million or even the billion-atom mark. These advancements in simulating larger systems have provided unprecedented opportunities to explore complex biological phenomena at an atomic level.

## Ligand pose prediction

Molecular docking programs are valuable CADD tools that predict the binding mode or “pose” of a small-molecule ligand within a specific binding-pocket conformation. Traditionally, pose prediction has relied on a single static protein structure derived from techniques such as X-ray crystallography. Although single-conformation docking can effectively identify true ligands, it fails to account for the possibility of alternative but equally valid pocket conformations. Moreover, many proteins lack co-crystallized ligands, making it challenging to experimentally determine their ligand-amenable *holo* states for use in CADD. In some cases, pharmacologically relevant cryptic pockets are entirely collapsed unless bound to a ligand. These limitations of single-conformation docking underscore the importance of methods that can better account for full protein dynamics.

In recent years, molecular docking studies have increasingly incorporated ensembles of diverse binding pocket conformations, often sourced from clustered MD simulations. Ensemble docking, also known as relaxed-complex-scheme docking, produces a spectrum of scores for each compound by docking each into multiple structures rather than just one. One then converts this spectrum into a single score, such as the ensemble-average or ensemble-best score, which is used to rank and prioritize compounds for experimental testing.

MD simulations have also emerged as valuable tools for validating docked poses. Though docking is widely used for drug discovery, the accuracy of docked poses is sometimes lacking. To address this shortcoming, researchers perform brief MD simulations of the predicted protein/ligand complex and monitor the ligand’s drift from its initial position. Correctly posed ligands tend to have greater stability, but incorrectly posed ligands often drift within the binding pocket.

## Predicting binding-free energies

MD simulations also play a crucial role in predicting ligand binding-free energies. Early-stage drug discovery aims not only to discover how small-molecule ligands interact with target proteins but also to assess their binding strength. Simulations provide valuable insights that help prioritize the most promising compounds for experimental validation and optimization. MM/GB(PB)SA and alchemical methods are popular MD-based approaches for evaluating binding-free energies.

MM/GB(PB)SA relies on one or more simulated trajectories of protein, ligand, and protein/ligand complexes. Selected frames from these simulations are used to calculate average binding-induced changes in molecular-mechanics and solvation energies. In recent years, machine learning has been used to improve the accuracy and efficiency of these methods by guiding simulation-frame selection [6], refining the calculation of MM/GBSA energy terms [7], and altering how the individual electrostatic, van der Waals, and solvation terms are combined into final free-energy estimates [8]. Machine learning has also been used to help researchers select the optimal number of MD and MM-PBSA runs to best strike a balance between accuracy and the need to conserve computational resources.

Though MM/GB(PB)SA methods are widely used, they consider only the bound and unbound states, neglecting the influence of intermediate states on binding energy. Alchemical simulation methods such as thermodynamic integration and free energy perturbation (FEP) [1] can overcome this challenge, albeit at a higher computational cost. These methods calculate relative free energies by gradually eliminating the nonbonded interactions between the ligand and environment (e.g., protein), effectively disappearing the ligand atoms during simulation. By observing the system’s response to these nonphysical changes, one can estimate the relative binding-free energies. Alchemical simulations have also benefitted from machine learning approaches. AlphaFold-predicted protein models are now often accurate enough to support FEP calculations [9], expanding the drug targets that can be studied using this technique. Machine learning can also reduce the number of alchemical calculations necessary to screen large chemical libraries in search of novel small-molecule inhibitors, an approach that has successfully identified inhibitors of various targets (e.g., the SARS-CoV-2 papain-like protease [10]).

## Conclusion

In recent decades, MD simulations and structure-based CADD have benefited from remarkable advancements in computational power, software development, and machine learning. Using these techniques, researchers can better capture the dynamics of ligand-binding pockets by simulating ever larger systems for ever longer timescales. The conformational ensembles these simulations reveal enable accurate ligand docking and binding-affinity prediction. As specialized hardware and algorithmic developments become increasingly accessible, the impact of MD simulations on early-stage drug discovery is poised to grow even further.

## Data Availability

Not applicable.
